# Atorvastatin in improvement of cognitive impairments caused by amyloid β in mice: involvement of inflammatory reaction

**DOI:** 10.1186/s12883-016-0533-3

**Published:** 2016-02-04

**Authors:** Liandong Zhao, Tingting Chen, Chonghui Wang, Guoxi Li, Wenhui Zhi, Jun Yin, Qi Wan, Ling Chen

**Affiliations:** Department of Neurology, The First Affiliated Hospital of Nanjing Medical University, 300 Guangzhou Road, Nanjing, Jiangsu 210029 China; Department of Neurology, The Second Hospital of Huaian, Huaian, Jiangsu 223002 China; Department of Physiology, Nanjing Medical University, Nanjing, 210029 China; Laboratory of Reproductive Medicine, Department of Physiology, Nanjing Medical University, 140 Hanzhong Road, Nanjing, China

**Keywords:** Alzheimer’s disease, Atorvastatin, Amyloid-β, Inflammatory, Long-term potentiation

## Abstract

**Background:**

The production of inflammatory cytokines resulting from amyloid β (Aβ) is associated with the initiation of Alzheimer’s disease (AD). Atorvastatin (ATV) has been reported to improve AD, however, it is unclear how the anti-inflammatory mechanism is linked with its protection against the impairment of spatial cognitive function in AD. The present study was designed to explore what mechanism was possibly involved in the anti-inflammatory pathway in regard to the ATV treatment of AD.

**Methods:**

We used an AD model induced by the administration of Aβ_25–35_ in male C57BL/6 mice and an in vitro culture system to study the protective effects of ATV on the spatial cognitive deficits, hippocampal long-term potentiation (LTP) impairment and inflammatory reaction.

**Results:**

The intragastric administration of ATV (5 mg/kg) in Aβ_25–35_-treated mice significantly ameliorated the spatial cognitive deficits and prevented the LTP impairment in hippocampal CA1. The increased Iba-1 positive cells and inflammatory components in the hippocampus were reduced after the ATV treatment. The anti-inflammatory and LTP protection of ATV were abolished using the replenishment of farnesyl pyrophosphate by the administration of farnesol (FOH). The hippocampal slices culture showed Aβ_25–35_-induced neurotoxicity in the absence of the presence of ATV. Treatment with ATV (0.5, 1, 2.5 μmol/L) dose-dependently prevented the cell damage in hippocampus induced by Aβ25–35.

**Conclusion:**

The administration of ATV ameliorated the cognitive deficits, depressed the inflammatory responses, improved the LTP impairment, and prevents Aβ25-35-induced neurotoxicity in cultured hippocampal neurons. These protective functions of ATV involved the pathway of reducing farnesyl pyrophosphate (FPP).

## Background

Alzheimer’s disease (AD) is the most common form of dementia, and is characterized by progressive loss of memory and cognition [1]. The initiation and progression of AD involve many different factors, such as Aβ42/Aβ40 ratio, elevation of cholesterol levels, oxidative stress, alterations in cholinergic nervous system and pro-inflammatory cytokines [2].

Amyloid β (Aβ) peptide has been shown to enhance microglial activation [3], and increase the production of inflammatory cytokines interleukin-1β (IL-1β), interleukin-6(IL-6) and tumor necrosis factor-α (TNF-α) [4–6]. Aβ 25–35 has been shown to have neurotoxic properties and to affect cognitive processes [7]. The short fragment represents the core functional domain of the full length Aβ peptide, and is able to self-assemble to form a predominantly β -sheet structure [8]. Therefore, our study used Aβ-25-35, instead of full-length Aβ, to establish the animal model of AD for studying neuro-toxic properties of AD and evaluations of anti-AD drugs [9]. The pro-inflammatory cytokines exacerbate the disease process, cause neuronal death [10], and disrupt synaptic function and induction of long-term potentiation (LTP) [11]. The anti-inflammatory agents have been reported to protect the Aβ-induced damages [12].

Atorvastatin (ATV), as a member of statins family, has been reported to decrease AD risk [11, 12]. Even though ATV is documented to attenuate the Aβ-induced inflammatory production, such as IL-1β, IL-6 and TNF-α, in the hippocampus [6], it is not fully clear whether the anti-inflammatory function is potentially linked with its protective effects on the spatial cognitive function. The present study, therefore, was designed and used farnesol (FOH), LY294002 and corticosterone (CORT) to examine whether the improvement of cognitive impairment by ATV-treatment involves the pathway of LTP induction in regard with the anti-inflammatory response following the administration of Aβ25-35 in animal model. In addition, an in vitro culture system was used to examine the toxicity of Aβ-peptide and the dose-dependent protective effects of ATV in cell cultures.

## Methods

### Animal allocation

The animals were handled according to the guidelines of the Care and Use of Laboratory Animals (NIH USA), and the Animals Scientific Procedures Act (UK).

The study was submitted to and approved by the Ethics Committee of Nanjing Medical University, China (NMU-103-2014).

**Fig. 1:**
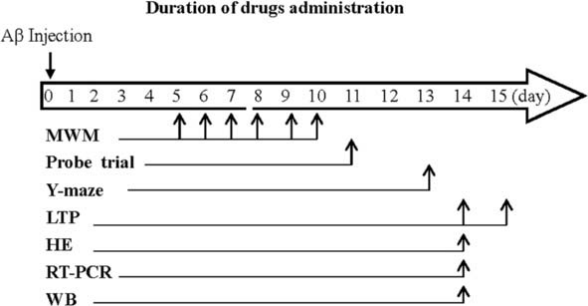
Time chart of experimental procedures. Hidden platform and probe trial test in Morris water maze (MWM) and Y-maze task was performed on days 5–11 and day 13 after Aβ_25–35_-injection, respectively. On day 14–15 after Aβ_25–35_-injection the mice were killed for electrophysiological recording LTP, histological examination (HE), Western blot (WB) and RT-PCR analysis. ATV, ATV + FOH, ATV + LY294002 (LY) or CORT were consecutively administered after Aβ_25–35_-injection. FOH or LY294002 was administered 30 min before ATV-treatment

Male C57BL/6 mice (Oirental Bio Service Inc., Nanjing), weighing 20–25 grams, were housed in a controlled room under a12-h light/dark cycle, and had free access to a standard pellet chow and tap water throughout the study. A total of ninety-six animals were randomly divided into the groups: Aβ (*n* = 8), Aβ + ATV (*n* = 8), Aβ + ATV + Farnesol (FOH) (*n* = 8), Aβ + ATV + LY294002 (*n* = 8), Aβ + Corticosterone (CORT) (*n* = 8), Aβ + CORT + ATV (*n* = 8) and their relevant controls (8 in each). The study was completed 15 days after the administration of Aβ_25–35_ (Fig. 1).

### Preparation of AD model

The AD model was prepared according to the previously reported method [6]. Briefly, Aβ_25–35_ (Sigma Chemical Co., St. Louis, MO, USA) was dissolved in a sterile bi-distilled water at a concentration of 2 mg/ml, and incubated at 37 °C for 4 days. 40 mg/kg of chloral hydrate was intraperitoneally administrated, and the animals were placed in a sterotactic device (Kopf Instruments, Tujunga, CA, USA). A volume of 3 μl of Aβ was injected into the right lateral ventricle, at the following coordinates at 3 mm posterior, 1 mm lateral and 2.5 mm ventral to bregma, using a stepper-motorized micro-syringe at a rate of 0.5 μl/min. The mice serving as the control group were administered with scrambled Aβ25–35 peptide (NeoMPS, Strasbourg, France) dissolved in a sterile bi-distilled water at the same volume.

### Drug administration

#### ATV

Four hours after the injection of Aβ_25–35_, the animals were intragastrically administered with ATV (Lipitor, Pfizer-Parke Davis, Ireland) mixing with food at a dose of 5 mg/kg per day. The animals were weighed at intervals, and the dose was accordingly adjusted throughout the study. The chosen doses of ATV was based on the previous studies [6].

#### Farnesol (FOH)

FOH (Sigma, Cat# 277541, St. Louis, MO, USA) was administrated by a gavage feeding or perfusion in slices. For the gavage feeding, FOH was administrated intragastrically at a dose of 100 mg/kg/day, as described previously [13, 14]. The intragastric administration of FOH started 30 min before ATV administration. The group serving as controls received saline only. For the perfusion in slices, following the previously described method [15], 1.85 μl of FOH was pipetted into 4 μL of 0.01 % ethanol, and then diluted into 40 ml of artificial cerebral spinal fluid to reach a final concentration of 2 μM. The control was infused with ethanol only.

#### LY294002

LY294002 (ApeBio, Houston, TX, USA), a potent inhibitor of phosphoinositide 3-kinases (PI3K), was prepared as the previously reported method [16]. Briefly, LY294002 was dissolved in dimethyl sulfoxide (DMSO), and mixed with saline to reach 1 % as a final concentration. LY294002 was intra-cerebroventricularly injected 30 min before the injection of ATV. For daily intra-cerebroventricular injection of LY294002, a 26-G stainless-steel guide cannula (Plastics One, Roanoke, VA, USA) was implanted into the right lateral ventricle and anchored to the skull with four stainless-steel screws and dental cement. The drug was injected using a stainless-steel needle combining with a stepper-motorized micro-syringe (Stoelting, Wood Dale, IL, USA) at a rate of 0.5 mL/min in a volume 3 mL/mouse. The control group were given the same volume of vehicle.

#### Corticosterone (CORT)

CORT (Sigma, St. Louis, MO, USA) was dissolved in ethanol (50 mg/mL), and diluted in sesame oil (8 mg/mL). Following the previously reported method [15], CORT was subcutaneously administrated at a dose of 40 mg/kg/day 30 min before the ATV administration. The controls received the same amount of ethanol/oil mixture.

### Y-maze task

The animals started with Y-maze task on day 13 after the administration of Aβ_25–35_. The Y-maze was performed as described previously [17]. Briefly, both the start arm (27.5 cm long) and two arms forming the Y (both 27.5 cm long and diverged at a 60° angle from the stem arm) were 5 cm in diameter. The home cage was connected to the start arm of the Y-maze. Each mouse was placed at the end of one arm, and allowed to move freely through the maze during an 8 min session. The series of arm entries was recorded visually, and arm entry was considered to be completed when the hind paws of the mouse were completely placed in the arm. Alternation was defined as successive entries into the three arms on overlapping triplet sets. The percentage alternation was calculated as the ratio of actual to possible alternations.

### Morris water maze

The animals started with Morris water maze on day 5 after the injection of Aβ_25–35_. A circular pool with diameter at 120 cm was prepared with the water temperature at 24 ± 1 °C. Ink powder was used to render the water opaque. Swim paths were analyzed using a computer system with a video camera (Neuroscience, Inc., Tokyo, Japan). The platform (7 cm in diameter) was submerged 1 cm below the water surface. Mice were given 90 s in the pool to search the hidden platform. If no platform was found within 90 s, the mouse was guided to the platform, and the trial was terminated. Each mouse started in one of four quadrants in a random manner, with the head facing the wall. Four trials were conducted each day, for six consecutive days. The probe trial was then recorded by removing the platform. The mouse was released from opposite quadrant in which the platform was located, and allowed to swim for 90 s to determine its search patterns. Times spent in platform quadrant, opposite quadrant, adjacent right and left quadrants were measured. The percentage of time spent in each quadrant was determined.

### Slice preparation and electrophysiology

Hippocampal slice preparation was performed as previously reported [18, 19] with modifications. Briefly, the animals were decapitated under deep anesthesia with ethyl ether (400 mg/kg, intraperitoneal injection), and the brains were rapidly removed out. Coronal slices (400 μM) of dorsal hippocampi were cut on a vibrating microtome (Dousaka EM Co, Kyoto, Japan) in an ice-cold cutting solution, and then incubated in artificial cerebrospinal fluid at 30 ± 1 °C for 60 min. After a slice was submerged in a recording chamber, hydraulic micromanipulators (Narishige, Tokyo, Japan) mounted on the microscopy were used to place a stimulating electrode in radiatum layer. Constant current pulses (0.1 ms, 0.06 Hz) were supplied by a stimulator (Nihon Kohden, Japan). The excitatory post-synaptic potential (EPSP) slope was recorded from radiatum layer with a 5 MΩ resistance glass microelectrode, and connected to a neutralized high input-impedance preamplifier. Stability of baseline recordings was established by delivering single pulses (four/min, 0.1 ms pulse width) for 15 min prior to collection of input/output functions. Baseline synaptic transmission was assessed by averaging the response to five pulses (from 0.1 to 1.0 mA) delivered at a rate of 0.06 Hz. Paired-pulse facilitation (PPF) was measured by using the intensity of the test stimulus with an inter-pulse interval of 25–100 ms. Pre-train responses were recorded for 20 min (baseline), high-frequency stimuli (100 Hz, 100 pulse) were used to induce LTP.

### Histological examination

Mice were anesthetized by intraperitoneal injection of chloral hydrate (40 mg/kg), and transcardially perfused with ice-cold phosphate-buffered saline followed by 4 % para-formaldehyde. The brains were quickly taken out, immersed in 4 % para-formaldehyde for fixation at 4 °C overnight, and processed for paraffin embedding. Coronal sections (5 μm) of hippocampus were prepared for the histological examination. The sections were treated with 3 % H_2_O_2_ for 10 min, and then incubated in 5 % goat serum for 30 min. For Iba-1 staining, the sections were incubated with a goat polyclonal anti-Iba-1 antibody (Abcam, Cambridge, UK), and then incubated in biotin-labeled anti-goat IgG antibody (Bioworld Technology, Inc., St. Louis Park, MN, USA) for 2 h at room temperature. The immunoreactivity was visualized by the standard avidin-biotin complex reaction with 3, 3′-diaminobenzidine (Vector Laboratories, Burlingame, CA, USA). Iba-1 positive cells were counted using a light microscope (Olympus, Japan). The density of Iba-1 positive cells was expressed as the mean number of Iba-1 positive cells per mm^2^.

### Hippocampal cell culture

Hippocampal slice cultures were prepared according to the previously reported method [20]. Briefly, mice were decapitated 15 days after Aβ25-35 administration. The brain was removed, and 400 μm of hippocampal slices were prepared, and separated in ice-cold HBSS solution with a pH value of 7.2. On average, six slices from the middle of hippocampus were obtained and placed in a 12-wells plate. The cells served as control group were from the animals without administration of Aβ25-35. The cell cultures were maintained in an incubator for 1 h before performing cell viability.

### Atorvastatin (ATV) preparation and cell treatments

The animals without Aβ25-35 administration were decapitated. Hippocampal slice cultures were prepared as reported previously [20]. To examine the dose-dependent protective effects of ATV, different doses at 0.5, 1 and 2.5 μmol/L in the culture medium were used. The cells cultures were maintained in an incubator with a 5 % CO_2_ mixed with 95 % O_2_ at 37 °C for 14 days, and the medium were replaced at every exchange of culture medium. The cells served as control group were treated with DMSO only in culture medium. The doses of ATV was chosen as described previously [21], which was confirmed to generate the dose-dependently inhibited effects.

### Quantification of cellular death

Cell death was assessed by using fluorescent exclusion dye propidium iodide (PI) uptake. PI is a polar compound that penetrates damaged cells only, and binds to nuclear DNA to generate a bright red fluorescence. The appearance of PI uptake is cellular membrane injury [22]. After 14 days of ATV exposure, 7 μm/ml of PI was added to the culture medium, and incubated for 40 min at 37 °C. Cultures were observed with an inverted microscope (Leica Microsystems Inc., Wetzlar, Germany) using a standard rhodamine filter set. The images were taken with an Olympus camera, and analyzed using Scion Image Software (http://scion-image.updatestar.com).

### Western blot analysis

Mice were decapitated under a deep anesthesia with chloral hydrate. Hippocampus was quickly taken out, and homogenized in a lysis buffer (Roche, Mannheim, Germany). Protein concentration was determined according to the instruction of the Protein Assay Kit (Pierce Biotechnology, Inc., Rockford, IL, USA). The membranes were incubated and developed by following the instruction of the ECL detection Kit (Millipore, Massachusetts, USA). Western blot bands were scanned and analyzed with the image analysis software package (NIH Image, Bethesda, MD, USA).

### Reverse transcription-polymerase chain reaction (RT-PCR)

Hippocampus was micro-dissected, and stored at −80 °C until assayed. RNA was isolated using Trizol reagent (Invitrogen, Camarillo, CA, USA), and reverse-transcribed into cDNA according to the instruction of Prime Script RT reagent kit (Takara Bio. Mountain View, CA., USA) for quantitative PCR in the presence of a fluorescent dye (Takara Bio. Mountain View, CA., USA). Relative expression of genes was determined using the reported method [23]. The levels of IL-1β and TNF-α mRNA were normalized by controls. The primers of IL-1β are 5′-CCATGGCACATTCTGTTCAAA-3′ and 5′-GCCCATCAGAGGCAAGGA-3′; the primers of TNF-α are 5′-ACGGCATGGATCTCAAAGAC-3′ and 5′-CGGACTCCGCAAAGTCTAAG-3′; the primers of GAPDH are 5′-ACCACAGTCCATGCCATCAC-3′ and 5′-TCCACCACCACCCTGTTGCTGTA-3′.

### Statistics analysis

The group data were expressed as the means ± standard error (SE). All statistical analyses were performed using SPSS software, version 16.0 (SPSS Inc., Chicago, IL, USA). Differences among means were analyzed using regressions analysis of variance. Differences at *P* < 0.05 were considered statistically significant.

## Results

### ATV improved spatial cognitive function impaired by Aβ_25–35_

**Fig. 2:**
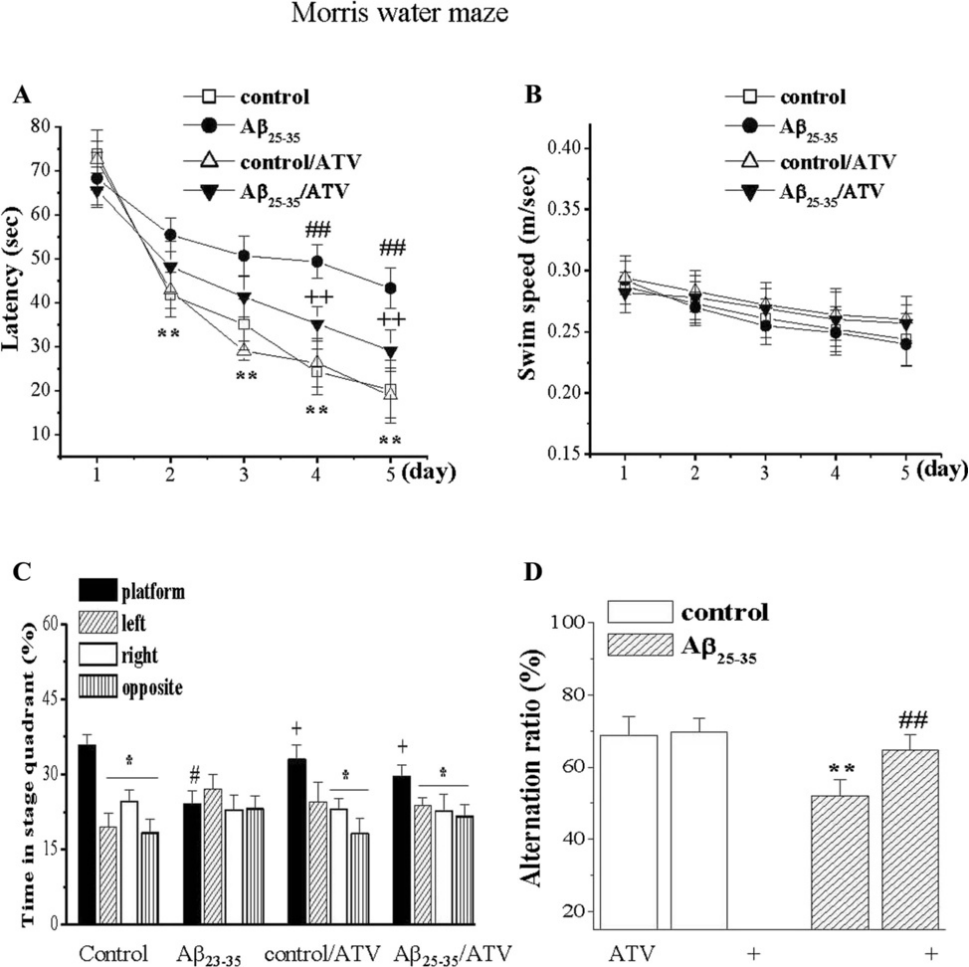
ATV ameliorates spatial cognitive deficits in Aβ_25–35_-mice. **a** Hidden-platform test in Morris water maze. Each point represents group mean latency (sec) to reach the hidden-platform in control mice (control), ATV-treated control mice (control/ATV, *n* = 8), Aβ_25–35_-mice (*n* = 8) and ATV-treated Aβ_25–35_-mice (Aβ_25–35_/ATV, *n* = 8). **P < 0.01 vs. latency on day 1 (comparisons within each group); ^##^ *P* < 0.01 vs. control mice; ^++^ *P* < 0.01 vs. Aβ_25–35_-mice (two-way ANOVA). **b** Swim speed (m/s) in Morris water maze. **c** In probe test, bars present the percentage of time spent in platform, opposite, right and left adjacent quadrants. *P < 0.05 vs. platform quadrant (comparisons within each group); ^#^ *P* < 0.05 vs. control mice; ^+^ *P* < 0.05 vs. Aβ_25–35_-mice (two-way ANOVA). **d** Bar graph shows group mean of alternation rate (%) in Y maze task. **P < 0.01 vs. control mice; ^##^ *P* < 0.01 vs. Aβ_25–35_-mice (two-way ANOVA)

Spatial cognitive performance was examined on day 5–11 after Aβ_25–35_-injection. The results from Morris water maze showed that the Aβ_25–35_-treated animals needed a longer time to find the hidden platform than those non-injection of Aβ_25–35_ (*P* < 0.01). The spatial cognitive impairment was significantly improved by the administration of ATV (*P* < 0.01). Even though no significant difference was observed in the swim speed, the swimming time spent in the platform quadrant was less in the Aβ-treated animals (*P* < 0.05). The decrease in the swimming time was improved after the administration of ATV. The results from the Y-maze test demonstrated that the administration of Aβ_25–35_ significantly decreased the spontaneous alternation behavior (*P* < 0.01), while the decrease was greatly reversed by the administration of ATV (*P* < 0.01). Those results suggested that ATV had anti-amnesic effects (Fig. 2a-d).

### ATV protected long-term potentiation (LTP) against impairment resulting from Aβ_25–35_

**Fig. 3:**
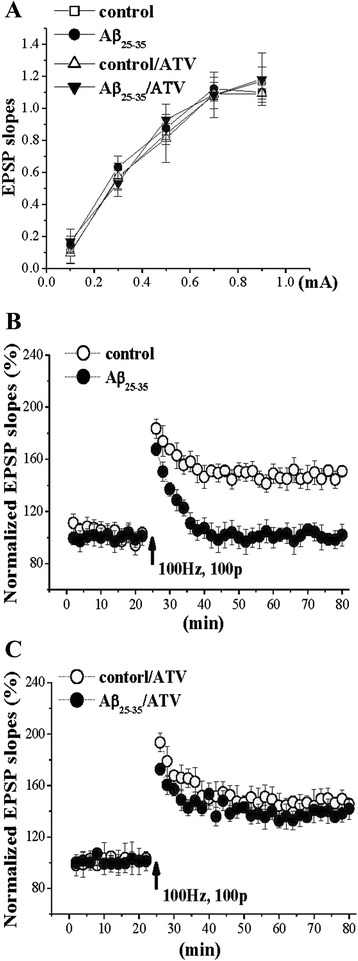
ATV rescues impairment of LTP induction in Aβ_25–35_-mice. **a** Input–output function at Schaffer collateral-CA1 synapses. EPSP slopes plotted against stimulus intensity (0.1–0.9 mA) in control mice (*n* = 8), ATV-treated control mice (control/ATV, *n* = 8), Aβ_25–35_-mice (*n* = 8) and ATV-treated Aβ_25–35_-mice (Aβ_25–35_/ATV, *n* = 8). **b** Induction of LTP by HFS (100 Hz, 100 pulses). Each point represents group mean EPSP slopes expressed as percentage of baseline. A solid arrow indicates when HFS was given. **c** Influence of ATV on Aβ_25–35_-impaired LTP induction

The slopes of excitatory postsynaptic potential (EPSP) using 0.1–0.9 mA stimuli did not difference in slices between the Aβ_25–35_-treated and control groups. A high-frequency stimulation (100 pulses at 100 Hz, 0.5 s in duration, 20 s interval) evoked an increase in EPSP slopes for over 60 min in the control group, but this increase was not seen in the Aβ-treated animals. The impairment of LTP caused by Aβ_25–35_ were fully reversed by the administration of ATV (Fig. 3a-c).

### ATV inhibited inflammatory responses resulting from the administration of Aβ_25–35_

**Fig. 4:**
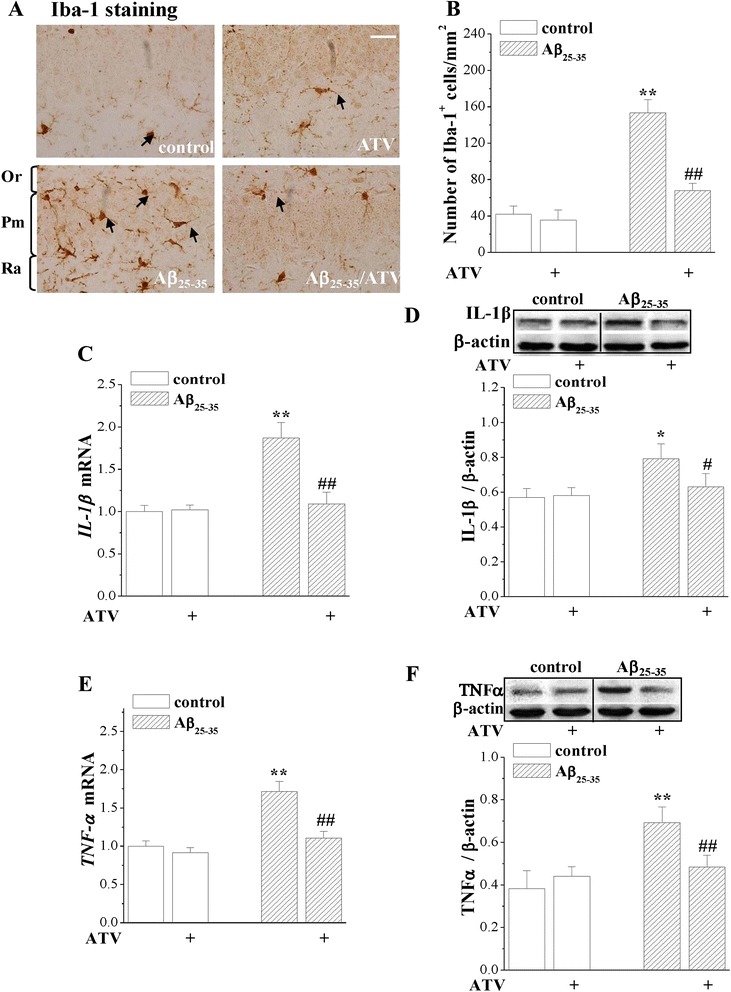
ATV inhibits the Aβ_25–35_-induced inflammatory responses in hippocampus. **a** Images of Iba-1 immunostaining in hippocampal CA1 of control mice (*n* = 8), ATV-treated control mice (control/ATV, *n* = 8), Aβ_25–35_-mice (*n* = 8) and ATV-treated Aβ_25–35_-mice (Aβ_25–35_/ATV, *n* = 8). Black arrows indicate Iba-1 positive cells. Scale bar = 50 μm. Or: oriens layer; Pm: pyramidal layer; Ra: radiatum layer. **b** Bar graphs show mean number of Iba-1 positive cells. **P < 0.01 vs. control mice; ^##^ *P* < 0.01 vs. Aβ_25–35_-mice (one-way ANOVA). **c & d** Bars present mean levels of *IL-1β* mRNA and IL-1β protein. **P < 0.01 and *P < 0.05 vs. control mice; ^##^ *P* < 0.01 and ^#^ *P* < 0.05 vs. Aβ_25–35_-mice. **e & f** Bar graph show mean levels of *TNF-α* mRNA and TNF-α protein. **P < 0.01 vs. control mice; ^##^ *P* < 0.01 vs. Aβ_25–35_-mice

The inflammatory production of IL-1β and TNF-α in the hippocampus were examined using the immunohistochemistry, Western blot and RT-PCR methods. The Aβ_25–35_ treatment caused a number of ionized calcium-binding adaptor molecule 1 (Iba-1) positive cells in pyramidal cell and radiatum layers, while the administration of ATV significantly prevented the responses (*P* < 0.01). Furthermore, the administration of Aβ_25–35_ resulted in elevations of IL-1β mRNA (*P* < 0.01), IL-1β protein (*P* < 0.05), TNF-α mRNA (*P* < 0.01) and TNF-α protein (*P* < 0.01), while the administration of ATV greatly attenuated those increases (Fig. 4a-f).

### FOH blocked the anti-amnesic and anti-inflammatory effects of ATV

**Fig. 5:**
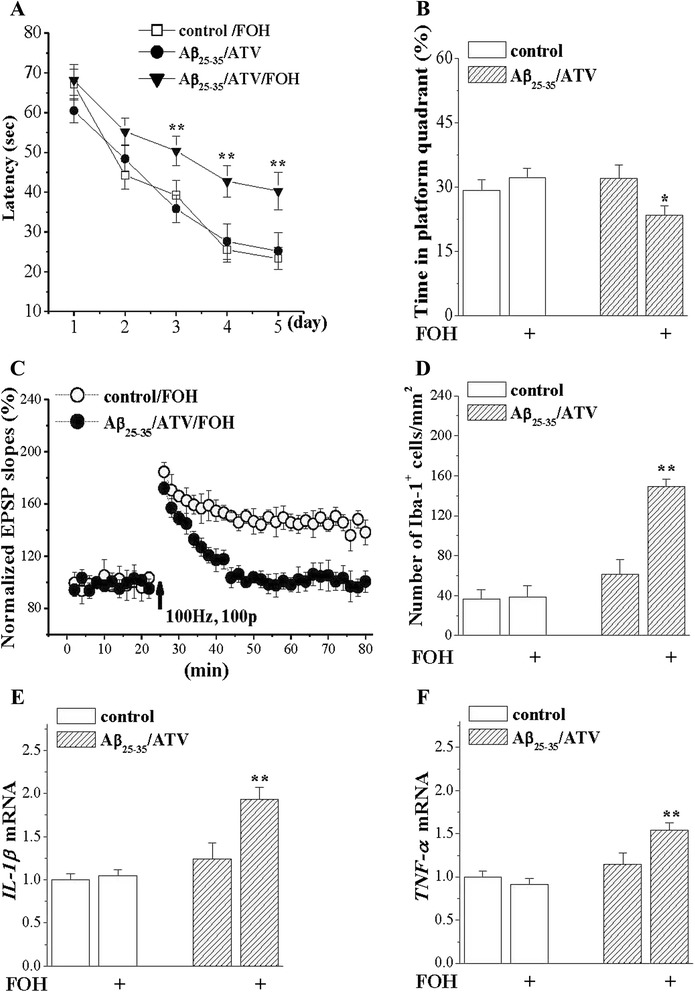
Anti-dementia, anti-inflammatory and LTP protection of ATV in Aβ_25–35_-mice are abolished by replenishment of FPP. **a** Each point represents group mean latency to reach hidden-platform in Morris water maze. **P < 0.01 vs. Aβ_25–35_/ATV. **b** In probe test, bars show the percentage of time spent in platform quadrant in Control, Control/ FOH, Aβ_25–35_/ATV and Aβ_25–35_/ATV/FOH mice (8 in each group). *P < 0.05 vs. Aβ_25–35_/ATV. **c** Induction of LTP in FOH-treated control mice (*n* = 8) and Aβ_25–35_/ATV mice (*n* = 8). **d** Bar graph shows mean number of Iba-1 positive cells in control mice (*n* = 8) and Aβ_25–35_/ATV mice treated with FOH (*n* = 8). **P < 0.01 vs. Aβ_25–35_/ATV. **e & f** Bar graphs show mean levels of *IL-1β* and *TNF-α* mRNA. **P < 0.01 vs. Aβ_25–35_/ATV

To test whether the anti-amnesic and anti-inflammatory effects of ATV were due to the reduction of farnesyl-pyrophosphate (FPP), we used FOH converting to FPP via endogenous salvage mechanisms. The results from the hidden platform task, probe test, and LTP induction showed that the administration of FOH blocked the protective effects of ATV on the Aβ_25–35_-impaired cognitive performance. In addition, the administration of FOH remarkably attenuated the inhibitory effects of ATV on the Aβ_25–35_-increased number of Iba1-positive cells (*P* < 0.01), the levels of IL-1β mRNA (*P* < 0.01) and TNF-α mRNA (*P* < 0.01, Fig. 5a-f).

### LY294002 inhibited the anti-amnesic and anti-inflammatory effects of ATV

**Fig. 6:**
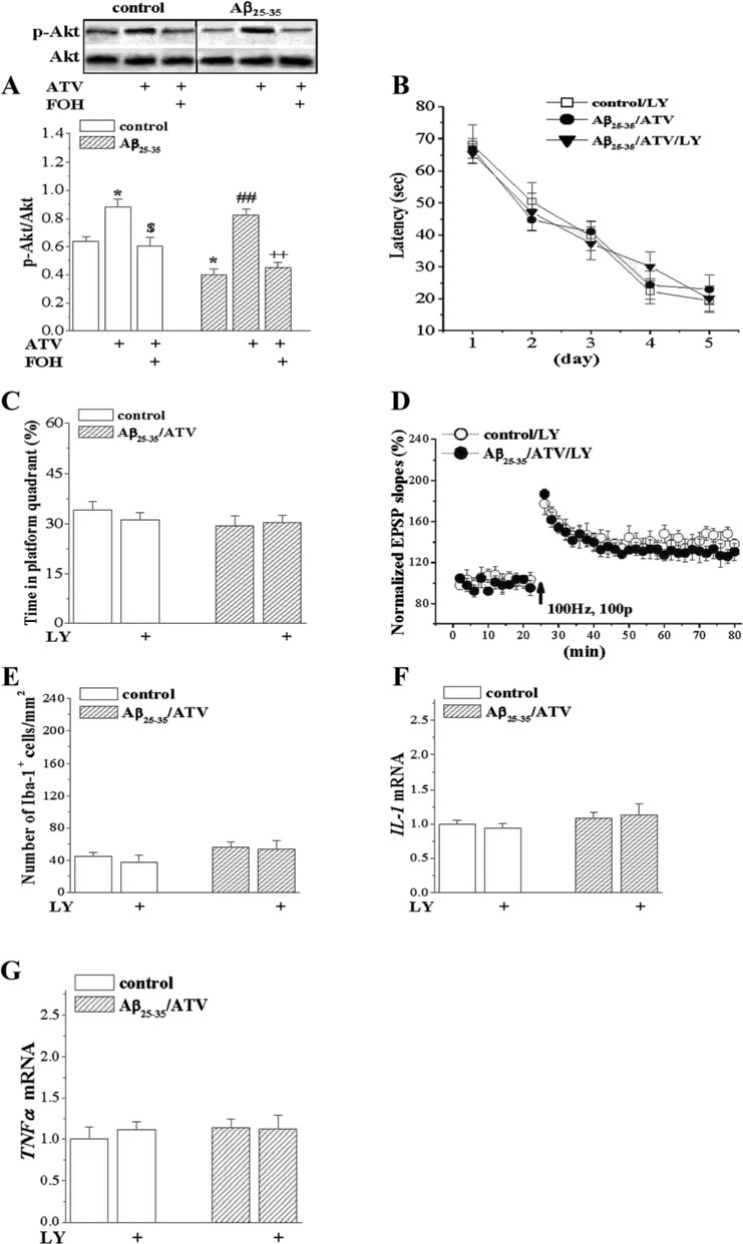
PI3K inhibitor had no effects on the anti-dementia, anti-inflammatory and LTP protection of ATV. **a** Western blot of phosphor-Akt in hippocampus in control and Aβ_25–35_ mice treated with ATV or FOH. *P < 0.05 vs. control mice; ^##^ *P* < 0.01 vs. Aβ_25–35_-mice; ^$^ *P* < 0.05 vs. control/ATV; ^++^ *P* < 0.01 vs. Aβ_25–35_/ATV (two-way ANOVA). **b**-**d** Effects of PI3K inhibitor LY294002 (LY) on the anti-dementia, anti-inflammatory and LTP protection of ATV in Aβ_25–35_-mice (*n* = 8). **e**-**g** The administration of LY294002, the effects of ATV on the microglial activation, and expressions of IL-1β and TNF-α in the Aβ25-35-treated animals were inhibited

LY294002 is an inhibitor of phosphoinositide 3-kinases (PI3K). ATV-enhanced LTP induction depends upon the PI3K/Akt activation [24]. Our results revealed that the administration of Aβ_25–35_ significantly decreased the level of Akt phosphorylation (phosphor-Akt) in the hippocampus (*P* < 0.05), the decrease was significantly recovered by the administration of ATV (*P* < 0.01). Moreover, the administration of LY294002 in the ATV-treated Aβ_25–35_-animals did not affect the spatial cognitive in hidden platform task, probe test, and LTP induction. Similarly, after the administration of LY294002, the effects of ATV on the microglial activation, and expressions of IL-1β and TNF-α in the Aβ_25–35_-treated animals were inhibited (Fig. 6a-g).

### Corticosterone (CORT) inhibited the inflammatory reaction and recovered LTP impairments causing from Aβ_25–35_

**Fig. 7:**
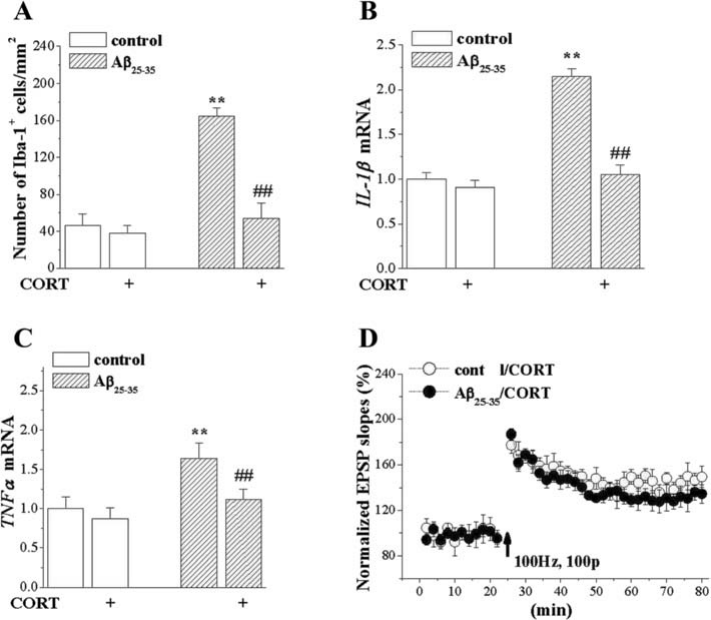
CORT inhibits anti-inflammatory and protects LTP protection in Aβ_25–35_-mice. **a** Bar graph shows mean number of Iba-1 positive cells in control mice (*n* = 8) and Aβ_25–35_ mice treated with CORT (*n* = 8). **P < 0.01 vs. control mice; ^##^ *P* < 0.01 vs. Aβ_25–35_ (two-way ANOVA). **b & c** Bar graphs show mean levels of *IL-1β* and *TNF-α* mRNA. **P < 0.01 vs. control mice; ^##^ *P* < 0.01 vs. Aβ_25–35_ (two-way ANOVA). **d** Induction of LTP in control mice (*n* = 8) and Aβ_25–35_ mice treated with CORT (*n* = 8)

To determine whether, through suppressing the Aβ_25–35_-induced inflammation, ATV protected the LTP induction and spatial memory. Our results showed that CORT decreased the number of Iba1-positive cells (*P* < 0.01), levels of IL-1β mRNA (*P* < 0.01) and TNF-α mRNA (*P* < 0.01). After the administration of CORT in the Aβ_25–35_-treated animals, the LTP induction was significantly recovered. These suggested that ATV protected the LTP induction and spatial memory was through the suppression of the Aβ_25–35_-induced inflammation (Fig. 7a-d).

### ATV protected cell death against Aβ25-35 induced damage

**Fig. 8:**
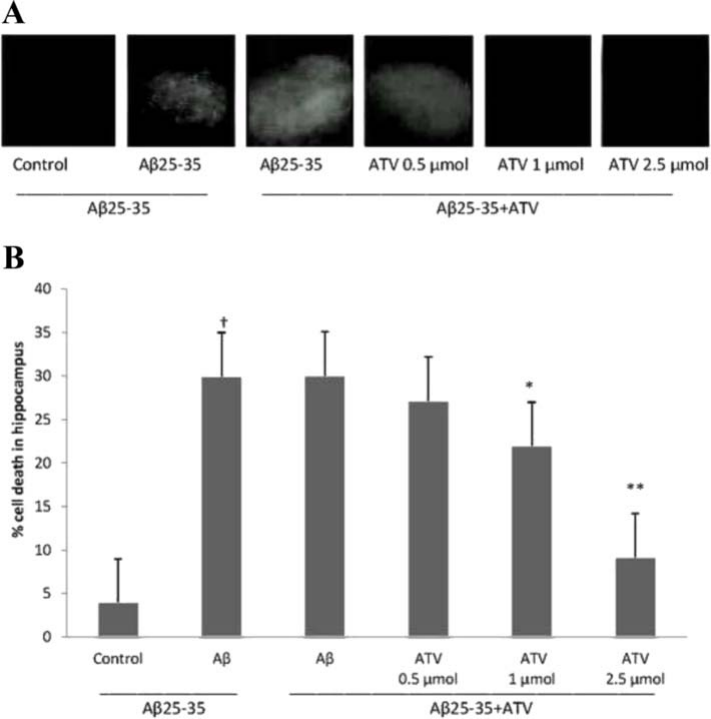
ATV attenuates cell damage after the pre-treatment of Aβ_25–35_ in hippocampal slices cultures. **a** Representative photomicrographs of PI uptake in hippocampal slices after the pretreatment with Aβ_25–35_ and exposure to ATV. **b** Quantification of PI uptake in response to Aβ_25–35_ and ATV. Values are expressed as percentage of cell death in hippocampus. †Compare with Control (*P* < 0.01), * compare with Aβ (*P* < 0.05), and ** compare with Aβ (*P* < 0.01). No significant difference was seen between Aβ and ATV-0.5 μmol groups

As expected, the cellular cultures from the animals treated with Aβ25-35 demonstrated a significant increase in fluorescence in hippocampal slices (*P* < 0.01), suggesting an increase in the cellular damage or death. These were significantly reduced by the treatment of 1 (*P* < 0.05) or 2.5 μmol/L (*P* < 0.01) of ATV, showing a dose-dependent protective effects on Aβ-induced cellular damage. Quantification of PI fluorescence showed that Aβ25-35 caused an increase in cellular damage (41 %) in the hippocampus, comparing with controls cultures (4 %). The treatment with 0.5 μmol/L of ATV did not protect from Aβ25-35 toxicity, however, when cells were treated with 1 and 2.5 μmol/L of ATV, the cell death was decreased to around 5 (1 μmol/L) and 15 % (2.5 μmol/L), respectively, suggesting a dose-dependent protection from the Aβ_25–35_-induced neurotoxicity (Fig. 8). The fluorescence reduction seen in the control group represents a minimal damage due to the slicing of hippocampus tissue.

## Discussion and conclusions

The neuroprotective mechanisms of statins therapy are a discussion topic in the medical literature. Studies suggest that the treatment with anti-inflammatory and cholesterol-lowering agents decreases the risk of developing AD. The most recent study also showed that the inflammatory factors were linked with the development of AD [6], but the study did not address what the exact mechanisms were involved. Our present study further confirmed that the administration of ATV greatly improved the spatial cognitive deficits caused by the administration of Aβ_25–35_, and demonstrated that the cognitive deficits resulting from Aβ_25–35_ involved inflammatory reaction.

Aβ has been shown to induce the production of pro-inflammatory cytokines IL-1β, TNF-α and IL-6 from microglia [5, 14, 25–27], and increase the activation of microglia, leading to the over-expression of IL-1β and TNF-α in hippocampus [28, 29]. The increases in these inflammatory components seem to link with the progression of AD [30]. Activated microglia release a diverse array of pro-inflammatory molecules that exacerbate the disease process and cause neuronal death [31]. The study by Boimel et al. [32] showed an anti-inflammatory or anti-microglial effect in ATV treated mice. The results from our study were consistent with those previously reported findings. In addition, a large number of Iba-1 positive cells was observed after the administration of Aβ_25–35_. Iba1 is a calcium-binding protein, and is specifically expressed in microglia in the brain. The administration of Aβ_25–35_ not only increased the number of Iba-1 positive cells, but also elevated the levels of IL-1β mRNA and IL-1β protein. However, the administration of ATV significantly attenuated the levels of those inflammatory components and the number of Iba-1 positive cells. These findings suggest that the pro-inflammatory responses attribute to the development of AD.

Isoprenoids, including farnesylpyrophosphate (FPP), serve as lipid attachments for all members of the small GTPase superfamilies [33]. Statins have been established to selectively reduce FPP, decreasing the membrane localization of Ras, Rho and Rab proteins. The inhibition of Rho-family function with Clostridium difficile Toxin A can reduce the inflammatory response [13]. The GTPases as molecular switches or timers can regulate critical cell-signaling pathways including those involved in inflammation response [34]. Our results showed that the ATV-increased Akt phosphorylation in Aβ_25–35_-treated animals seemed not associate with the anti-inflammatory effects, as the administration of Aβ_25–35_ decreased the level of phosphor-Akt, while ATV significantly recovered the alteration of phosphor-Akt level. Therefore, the Aβ_25–35_-induced inflammatory response inhibited by ATV is through the pathway of reducing FPP, and the anti-inflammatory effects can be blocked by the replenishment of FPP. However, further studies are needed to identify how the ATV anti-inflammatory mechanisms are involved in the pathway of reducing FPP.

In addition to increase the pro-inflammatory cytokines associated with the deficits in LTP induction and spatial cognition after administration of Aβ, our finding demonstrated that the spatial memory deficits were improved by the administration of ATV. The improvements were obviously through the anti-inflammatory pathway, as addressed early in the text, suggesting that the pro-inflammatory cytokines were increased through postsynaptic mechanisms, which impairs the LTP induction leading to spatial cognitive deficits. These findings also suggested that a modulation of hippocampal LTP was one of the underlying cellular and molecular mechanisms by which ATV treatment enhanced learning and memory [32].

The mevalonate pathway produces a number of intermediate compounds including FPP associated with the control of several cell functions through protein prenylation [35]. In this experimental model as a consequence of an inflammatory stimuli produced by Aβ injection, a consumption of mevalonate isoprenoids occurs. In normal conditions, the isoprenoids are continuously replaced through the mevalonate pathway, allowing a good feedback control of inflammation and a sufficient availability of intermediates for the synthesis of cholesterol. However, in the presence of mevalonate pathway deficiencies, the supply of anti-inflammatory isoprenoids is inadequate, leading to an inflammatory reaction [36]. To examine if the anti-inflammatory effects of ATV are associated with a reduction of isoprenoid intermediates, we employed FOH in the Aβ_25–35_-treated animals. The administration of the exogenous isoprenoids FOH, that have been already shown to reduce inflammatory parameters in an animal model [36]. The results of the present study are in conformity with the reported findings. Our results showed that the inhibitory effects of ATV on the Aβ_25–35_-increased number of Iba1-positive cells and pro-inflammatory cytokines were attenuated, and the protective effects of ATV on the Aβ_25–35_-impaired cognitive performance were blocked by FOH, suggesting that FOH exerted as a modulator of LTP induction in area CA1.

The steroid hormone corticosterone (CORT) is released from the adrenal glands. In the brain, there are the cellular and molecular targets for the action of CORT. The regulation of LPT by CORT in the hippocampus has been reported in the previous studies [37]. In order to determine whether ATV protected the LTP induction and spatial memory was through suppressing the Aβ_25–35_-induced inflammation, our study used CORT in the Aβ_25–35_-treated animals, and our results showed that CORT had anti-inflammatory effects associated with a recovery of LTP induction. These confirmed that protective effects of ATV on the LTP induction and spatial memory were through suppression of the Aβ_25–35_-induced inflammation.

ATV-enhanced LTP depends upon PI3K/Akt activation [26]. The level of phosphor-Akt in hippocampus of mice was decreased by the administration of Aβ_25–35,_ which was recovered by the ATV-treatment. The ATV-increased phosphor-Akt was blocked by FOH. However, the administration of LY294002 did not affect the spatial cognitive, probe test and LTP induction. The inhibitory effects of ATV on the microglial activation and the expression of IL-1β and TNF-α in Aβ_25–35_-mice were insensitive to the administration of LY294002. Our present data, therefore, proposed that the protections of ATV is through a suppression of the pro-inflammatory cytokines resulting from Aβ_25–35_.

On the other hand, the pro-inflammatory cytokines may link with neuronal dysfunction and cell death induced byAβ25–35 peptide. In this study, we observed a significantly increased cell damage or death when cells were pretreated with Aβ25–35. To examine whether ATV is protective for the cell damage or death, we used ATV administrated in vitro at different dosages. The findings demonstrated that the cell damage were greatly prevented by the ATV treatment with dose-dependently inhibited effect. In addition, the results from our in vitro study showed that the paradigm of cellular damage caused by Aβ25–35 and the protection of ATV treatment were the same with those observed in animal model. A recent study by Piermartiri et al. [29] has also indicated that ATV prevents hippocampal cell death and neuro-inflammation following Aβ administration, and concluded that the mechanisms of those actions involve different pathways.

The overall results of our work suggest that Aβ_25–35_ results in spatial cognitive deficits associated with pro-inflammatory responses and LTP impairment, and that ATV treatment protects these impairments against the anti-inflammatory effects through a reduction of the FPP production. However, these improvements may also link with other changes in the circulation, vascular permeability and cholesterol-lowering following the administration of ATV. Therefore, the further studies are extremely needed.

### Limitation of the study

A cell culture experiment would help to clarify if ATV treatment reduces the secretion of pro-inflammatory cytokines. However, the results from our study showed that the paradigms of cellular death caused by Aβ25–35 and ATV protection observed in vitro culture were similar with those in animal model. In addition, the inflammatory cytokines associated with the ATV neuro-protection following Aβ administration has been evidenced in the recent study [29].
